# Inhibition of nuclear factor kappaB proteins-platinated DNA interactions correlates with cytotoxic effectiveness of the platinum complexes

**DOI:** 10.1038/srep28474

**Published:** 2016-08-30

**Authors:** Viktor Brabec, Jana Kasparkova, Hana Kostrhunova, Nicholas P. Farrell

**Affiliations:** 1 Institute of Biophysics, Academy of Sciences of the Czech Republic, v.v.i., Kralovopolska 135, CZ-61265 Brno, Czech Republic; 2 Department of Biophysics, Faculty of Science, Palacky University, Slechtitelu 27, CZ-78371 Olomouc, Czech Republic; 3 Department of Chemistry, Virginia Commonwealth University, Richmond, VA 23284-2006, USA

## Abstract

Nuclear DNA is the target responsible for anticancer activity of platinum anticancer drugs. Their activity is mediated by altered signals related to programmed cell death and the activation of various signaling pathways. An example is activation of nuclear factor kappaB (NF-κB). Binding of NF-κB proteins to their consensus sequences in DNA (κB sites) is the key biochemical activity responsible for the biological functions of NF-κB. Using gel-mobility-shift assays and surface plasmon resonance spectroscopy we examined the interactions of NF-κB proteins with oligodeoxyribonucleotide duplexes containing κB site damaged by DNA adducts of three platinum complexes. These complexes markedly differed in their toxic effects in tumor cells and comprised highly cytotoxic trinuclear platinum(II) complex BBR3464, less cytotoxic conventional cisplatin and ineffective transplatin. The results indicate that structurally different DNA adducts of these platinum complexes exhibit a different efficiency to affect the affinity of the platinated DNA (κB sites) to NF-κB proteins. Our results support the hypothesis that structural perturbations induced in DNA by platinum(II) complexes correlate with their higher efficiency to inhibit binding of NF-κB proteins to their κB sites and cytotoxicity as well. However, the full generalization of this hypothesis will require to evaluate a larger series of platinum(II) complexes.

*cis*-Diamminedichloridoplatinum(II) (cisplatin) is one of the most potent antitumor agents in cancer chemotherapy^1^. It is generally accepted that the cytotoxic activity of cisplatin and other platinum antitumor drugs results from their interactions with DNA^2^,^3^. However, many tumor cells display inherent or acquired resistance to platinum-based drugs, which further limits their utility^4^. Multiple signaling pathways have been linked to tumor resistance to cisplatin, among them also activation of nuclear transcription factor kappaB (NF-κB)^5^. Interestingly, suppression of apoptosis or necrosis is an important NF-κB function^6^,^7^.

Sequence-specific DNA binding is one of the key biochemical activities responsible for much of the biological functions of NF-κB^8^,^9^. Furthermore, it has been reported^10^ that cisplatin adducts formed in the DNA consensus sequence (κB site) reduce its binding affinity to NF-κB proteins, which may affect these key biochemical activities. In contrast, the affinity of NF-κB to the κB sites is not affected by the adducts of clinically ineffective transplatin. In addition, thousands of κB sites are present in the natural DNAs^11^,^12^ and interestingly, these κB sites conserve the consecutive guanines^13^,^14^ which represent preferential DNA binding sites of antitumor platinum(II) complexes. Hence, it has been suggested that the reduced affinity of the NF-κB proteins to the κB sites as a result of their modification by cisplatin is relevant to the biological activity of this drug.

This work, which is an extension of the previous study^10^, attempts to verify the hypothesis and support the view that mechanisms underlying toxicity of bifunctional platinum(II) complexes in tumor cells are connected with efficiency of these metal complexes to inhibit binding of NF-κB to its DNA consensus sequence (κB site). The need to further verify this previously proposed hypothesis emerges from the fact that it has been only based on comparisons of the effects of two platinum(II) complexes, namely antitumor cisplatin and its clinically ineffective trans isomer (transplatin). Therefore, we used for this study another bifunctional platinum(II) agent with a unique mode of DNA binding, the trinuclear [{*trans*-PtCl(NH_3_)_2_}_2_(μ-*trans*-Pt(NH_3_)_2_{NH_2_(CH_2_)_6_NH_2_}_2_)]^4+^ (Triplatin, BBR3464) (Fig. 1). This complex is markedly more cytotoxic than cisplatin and its antitumor derivatives used in the clinic^15^,^16^,^17^ and retains activity against cell lines and tumors resistant to cisplatin *in vitro* as well as *in vivo*^18^,^19^. DNA adducts of BBR3464 are distinctly different from those of the mononuclear cisplatin and its antitumor derivatives. In contrast to cisplatin, which forms on DNA major 1,2-GG or AG intrastrand cross-links (CLs)^20^, BBR3464 forms on DNA long range intra or interstrand CLs, i.e. CLs formed preferentially between guanine residues separated by one or more intervening base pairs. The distortions induced in DNA conformation by these long range CLs are different from those induced by the CLs of cisplatin and extend over more base pairs^21^,^22^,^23^. Directional isomers, where interstrand CLs are formed in the “normal” 5′ ->5′ direction as well as the converse 3′->3′ are also formed^23^. Thus, we examined whether the structurally distinct family of BBR3464-DNA adducts perturb the кB site–NF-κB protein interaction and compared the results with those of identical experiments performed with cisplatin or transplatin. In the present study, the binding properties of NF-κB reconstituted from purified p50 and p65 proteins and the native complex of NF-κB to DNA containing κB site damaged by DNA adducts of BBR3464 was investigated with the aid of classical electrophoretic mobility shift assay (EMSA) and surface plasmon resonance (SPR) spectroscopy which makes it possible to study such interactions in real time as well.

## Results

### Binding of recombinant NF-κB proteins to DNA modified by BBR3464 containing the κB consensus response element

In these experiments an oligonucleotide duplex 22 bp-long (Fig. 2A) incorporating centrally located consensus sequence 5′-GGGACTTTCC/5′-GGAAAGTCCC (DUPLEX-κB, shown in bold in Fig. 2A) was used. This sequence belongs to the immunoglobulin κ light chain gene enhancer (Ig-κB) which is present in many important genes responsive to NF-κB^13^. The DUPLEX-κB was globally modified by BBR3464 as described in Materials and Methods at r_b_ = 0.023, 0.045, or 0.091 [r_b_ is defined as the number of molecules (not platinum atoms) of the platinum complex bound per nucleotide residue]. The samples containing these duplexes were incubated with NF-κB proteins (either p50/p50, p65/p65 homodimers or p50/p65 heterodimer) at protein to duplex molar ratio of 10. To avoid nonspecific interactions, an excess of unplatinated synthetic double-helical poly(dI-dC).poly(dI-dC) was also added to the reactions as the nonspecific competitor. Samples were then analyzed by native PAA gel electrophoresis (PAGE) (the panels on the left in Fig. 2B–D).

Incubation of the unplatinated DUPLEX-κB with NF-κB proteins resulted in a new, more slowly migrating band, with a concomitant decrease of the intensity of the band corresponding to the free DUPLEX-κB (the panels on the left in Fig. 2B–D, lanes 1 and 2), confirming formation of the complex NF-κB-DUPLEX-κB. Interestingly, the incubation of the DUPLEX-κB modified by BBR3464 with p50/p65 heterodimer also resulted in formation of DNA-protein complexes. However, the intensities of the bands corresponding to these complexes were significantly reduced compared to intensity found for the band corresponding to nonplatinated oligonucleotide probe (left panel in Fig. 2B). This effect was more pronounced with growing level of platination of DUPLEX-κB. Similar results were obtained also for both p65/p65 and p50/p50 homodimers (left panels in Fig. 2C,D). These results indicate that DNA adducts formed by BBR3464 effectively inhibit binding of NF-κB proteins to the recognition sequence. Purified platinated oligonucleotide probes were used in these experiments so that the reactions were performed in the absence of any free platinum complex in the incubation mixtures. Hence, the observed effects could not be affected by the binding of free molecules of the BBR3464 to the proteins.

Inhibition of the formation of the complex between NF-κB proteins (p50/p65, p65/p65 or p50/p50) and the DUPLEX-κB globally modified by BBR3464 and by cisplatin or clinically ineffective transplatin determined previously under identical experimental conditions^10^ was compared (right panels in Fig. 2B–D). The trend is BBR3464 > cisplatin > transplatin confirming that the structurally diverse family of BBR3464-DNA adducts are most effective in inhibiting κB DNA- NF-κB protein formation.

### Binding of native complex of NF-κB to DNA modified by BBR3464 containing the κB consensus site

The experiments described in the preceding section were performed with purified recombinant proteins p50 and p65 in a cell-free medium. To correlate with cellular behavior, binding of native NF-κB present in whole cell extract to DNA containing the Ig-κB site modified by BBR3464 was also examined. Native NF-κB proteins present in nuclear extracts consist of both Rel and non-Rel subunits that comprise multiple protein complexes with different gene activation specificities^24^,^25^. In the following experiments, whole cell extracts from cytokine interleukin-1 alpha (IL-1α)-stimulated HeLa cells were used. IL-1α causes activation of the NF-κB signal transduction pathway. Incubation of the unplatinated DUPLEX-κB with the IL-1α stimulated cell extract resulted in formation of DNA-protein complexes. These complexes appeared on the gel as a new, more slowly migrating species (Fig. 3A, lane 2). Concomitantly, a decrease in the intensity of the band corresponding to the free duplex was observed (Fig. 3A, lane 2).

This result can be interpreted to mean that the complex between oligonucleotide duplex containing consensus site and native NF-κB proteins was formed. The fact that identical, but cold (unlabeled) oligonucleotide duplex was able to compete away the induced bands confirmed the specificity of these bands. Moreover, an additional shift was observed when antibodies against the p50 and p65 subunits of NF-κB were added, which also confirmed the specificity of the bands corresponding to the DNA-protein complexes. The changes in mobility also indicated that the upper two more slowly migrating bands contained p65/p65 homodimer and p50/p65 heterodimer consistent with previous reports^24^,^26^.

The global modification of the oligonucleotide probe by BBR3464 at r_b_ = 0.023, 0.045 or 0.091 reduced the yield of the DNA-protein complexes formed as a results of incubation with the whole cell extract stimulated by IL-1α (Fig. 3A, lanes 3–5 and Fig. 3B). The modification of oligonucleotide probe containing κB site at r_b_ = 0.091 completely inhibited formation of the complex between this duplex and native NF-κB proteins (Fig. 3A, lane 5 and Fig. 3B). Thus, the results of these experiments corroborated a strong inhibitory effect of DNA adducts of BBR3464 on the binding affinity of native NF-κB proteins to the κB consensus site.

Inhibition of the formation of the complex between native NF-κB proteins and the 22-bp oligonucleotide duplex containing consensus sequence (DUPLEX-κB) globally modified by BBR3464 and by cisplatin or clinically ineffective transplatin determined previously under identical experimental conditions^10^ was compared (Fig. 3B). The trend is again as observed in the cell-free medium: BBR3464 > cisplatin > transplatin.

### Binding of NF-κB to platinated DNA lacking the recognition κB site

Further experiments were aimed at examining binding of NF-κB proteins to the 22-bp oligonucleotide duplex lacking the consensus κB site (Scrambled DUPLEX, see Fig. 4A), which was modified by BBR3464 under identical experimental conditions as described in the preceding paragraphs. No changes in the migration of the duplex lacking the κB site were observed as a consequence of its incubation with p50/p50 or p50/p65. This result indicates that complex between NF-κB proteins and DNA lacking κB site was not formed (Fig. 4B,C lanes 3–5). Thus, these results demonstrate that DNA adducts of BBR3464 do not represent a structural motif which is recognized by NF-κB proteins.

### Binding kinetics and affinities using Surface plasmon resonance (SPR) spectroscopy

To delineate more precisely the binding properties of NF-κB proteins with DNA containing κB-sites modified by platinum complexes including information on the dynamics of binding of NF-κB proteins, we have carried out binding studies using *Surface plasmon resonance* (SPR) technology. SPR spectroscopy makes possible real-time detection and the determination of binding kinetics and affinities of biomacromolecular interactions^27^,^28^.

We studied binding of p50/p50 homodimer, as a representative of NF-κB proteins, which binds specifically to sites recognized by NF-κB^29^, to biotinylated oligonucleotide constructs [DUPLEX-κB(SPR-BIO) and Scrambled DUPLEX(SPR-BIO), Fig. 5B)]; these duplexes were unplatinated or globally modified by platinum complexes. These duplexes used for SPR experiments were similar to those used in EMSA except that a biotin residue was added to the 3′ end of the bottom strand for binding to the Biacore streptavidin chip.

The biotin-labeled DNA duplexes [DUPLEX-κB(SPR-BIO) or Scrambled DUPLEX(SPR-BIO)] were first immobilized on the surface of SA chip which was coated with streptavidin. The p50/p50 homodimer samples were then injected and flowed through the channels on the chip and interacted with the bound duplexes. The details of these procedures are described in the section Materials and methods. Upon injection through the SPR flow cell, the protein solution is replenished within the system so the protein concentration effectively remains constant at the initial value. When the association phase is long enough the binding reaction reaches equilibrium. There is a linear relationship between the amount of bound material and response units (RU).

The typical sensorgrams are shown in Fig. 6. They were exemplified by an abrupt response followed by a steady increase until the plateau was reached. The association sensorgram of each sample was recorded for 400 s followed by 200 s of running buffer injection period for dissociation. The protein concentrations ranged from 10 to 200 nM. By comparing binding to duplexes containing κB site and scrambled sequence [DUPLEX-κB(SPR-BIO) and Scrambled DUPLEX(SPR-BIO), respectively] it was verified that no noticeable nonspecific interactions between DUPLEX-κB(SPR-BIO) and NF-κB protein occurred under these conditions.

The sensorgrams were analyzed by BIAevaluation 3.1 software. After blank subtraction, values of response units (RU) at 400 s were read and plotted against protein concentration (Fig. 7A). At protein concentration of 100 nM plateau level was reached for all samples.

Platination of DUPLEX-κB(SPR-BIO) resulted in a decrease of the plateau level indicating inhibition of the formation of the complex between p50/p50 homodimer and DUPLEX-κB(SPR-BIO). This decrease was most pronounced when the duplex was modified by BBR3464 (decrease to 21%) whereas modification of the duplex by ineffective transplatin was only small (decrease to only 88%) (Fig. 7B). Thus, the trend in the efficiency of the platinum complexes tested in this work to inhibit formation of the complex between DNA duplex containing κB-site and the NF-κB protein was BBR3464 > cisplatin > transplatin. It implies that the results of steady-state SPR spectroscopic analyses were fully consistent with and confirmed the conclusions based on the results of EMSA (Figs 2 and 3).

Binding of p50/p50 homodimer to the Scrambled DUPLEX(SPR-BIO) (lacking the consensus κB site) which was modified by BBR3464, cisplatin or transplatin under identical experimental conditions as described for DUPLEX-κB(SPR-BIO) was examined as well. After immobilizing the biotinylated duplex Scrambled DUPLEX(SPR-BIO) on the surface of SA chip coated with streptavidin, p50/p50 homodimer was injected over the surfaces. No increase of RU typical for the association sensorgrams was noticed, which indicates that complex between the NF-κB protein and DNA duplex lacking κB site was not formed. Thus, also these results of SPR spectroscopic analysis demonstrate that adducts of BBR3464 formed on DNA do not represent a structural motif which is recognized by NF-κB proteins.

The SPR sensorgrams were further analyzed to estimate the association and dissociation rate constants as well as the dissociation equilibrium constant *K*_D_ and Gibbs free energy change (Δ*G*^0^_310_) (Table 1). Inspection of the thermodynamic parameters *K*_D_ and Δ*G*^0^_310_ revealed that the modification of DUPLEX-κB(SPR-BIO) by platinum complexes tested in this work reduced the duplex thermodynamic stability. The efficiency of DNA adducts formed by transplatin, cisplatin and BBR3464 to reduce the thermodynamic stability differed; the trend was transplatin < cisplatin < BBR3464.

Examination of the association and dissociation phases of SPR sensorgrams shows that the kinetic parameters responsible for the affinity decrease are different for duplexes containing κB sites [DUPLEXes-κB(SPR-BIO)] and modified by transplatin, cisplatin or BBR3464 Table 1). This decrease related mainly to variation of the association step, p50/p50 homodimer association was slowest to the duplex modified by BBR3464 and when DUPLEX-κB(SPR-BIO) was modified with transplatin, the association rate was reduced the least.

### Binding of NF-κB to purified κB–site containing oligonucleotide carrying single platinum adduct in the absence of unplatinated duplexes

The oligonucleotide duplexes used in the EMSA experiments described in Figs 2 and 3 (DUPLEXes-κB) were globally modified by the platinum complexes at r_b_ = 0.023, 0.045, or 0.091, i.e. 2.3, 4.5, or 9.1 molecules of the platinum complex was bound per 100 base residues in average. Considering a probability of distribution of the platinum molecules bound to the duplex, the samples used for these experiments could contain also a fraction of unplatinated molecules (as well as a certain fraction of oligonucleotide molecules bearing more than one platinum adduct), which could affect the resulting response. Therefore, the sample of the oligonucleotide duplex (DUPLEX-κB) containing a single adduct of BBR3464 and no unplatinated duplexes was prepared and tested for its inhibitory effect. The bottom strand of κB-site containing oligonucleotide duplex (DUPLEX-κB, for its nucleotide sequence, see Fig. 2A) was reacted with equimolar concentration of BBR3464 in NaClO_4_ (10 mM) in the dark for 24 h. The platinated oligonucleotide was purified by HPLC.

The HPLC profile of bottom strand of κB-site containing oligonucleotide is shown in Fig. 8, curve 2. This profile contains three peaks labeled X, 3Pt, and NoPt. The peak NoPt appeared at the same retention time as the peak corresponding to the unplatinated bottom strand of the oligonucleotide (curve 1) so that it was assigned to the unplatinated strand. The products corresponding to the peaks 3Pt was collected. Flameless atomic absorption spectrophotometry (FAAS) and optical density measurements were used to verify that the modified oligonucleotide contained one molecule of BBR3464 (3 platinum atoms) per one strand. This platinated bottom strand was allowed to anneal with the complementary top strand which was radioactively labeled on the 5′-end in NaClO_4_ (0.1 M). The resulting duplex was further purified on 12% native PAA gel so that the sample only encompassed oligonucleotide duplexes which contained just 1 adduct of BBR3464 (and no non-annealed single strands). On the other hand, it cannot be excluded that these samples also contained a small fraction of duplexes in which the single adduct was formed outside the κB site. An EMSA experiment was performed with this sample under the same experimental condition as described for globally modified oligonucleotides; concentration of the oligonucleotide probe was 1 nM and the concentration of p50/p50 was 10 nM. For other details, see the section Materials and methods.

Similarly to the globally modified κB-site containing oligonucleotide (Fig. 2), presence of just one adducts of BBR3464 inhibited formation of the complex between this duplex and p50/p50 homodimer (Fig. 2B). However, this inhibition was markedly higher than that observed when the same oligonucleotide was globally modified by BBR3464 to the same level of modification. While a global modification of κB-site containing probe reduced amount of DNA/protein complex by 70%, (Fig. 2B), the removal of the unplatinated duplexes resulted in the complete inhibition (Fig. 8B). Qualitatively identical results were also obtained with p50/p50 or p65/p65 homodimers. This result suggested that presence of a portion of unplatinated duplexes very likely lowered the inhibition effect of the global platination on the formation of the complex between this duplex and NF-κB proteins (Figs 2 and 3).

### Binding study of NF-κB to the platinated DNA κB sites in cells

To determine the effect of BBR3464 modification of the кB site on the DNA binding activity of NF-кB proteins in cells, the decoy strategy has been employed as already described in our recent study^10^. We used a decoy oligonucleotides of dumbbell shaped structure (Fig. 9A). These vectors are composed of a linear double-stranded stem parts containing the κB consensus sequence (DUMBBELL-κB) or scrambled sequence (Scrambled DUMBBELL) which are covalently closed at both ends with single-stranded loops. This structure improves resistance of oligonucleotides to cellular nucleases as a result of a lack of free ends.

In order to study the effect of BBR3464 on the decoy activity of DUMBBELL-κB, human embryonic kidney (HEK)293 cell line containing a stably integrated luciferase-reporter gene driven by a NF-кB-responsive promoter with multiple κB elements was transfected with platinated or nonplatinated dumbbells and luciferase expression was measured in these transfected cells. The specific inhibition of transcriptional activity of NF-кB in HEK293 cells by the DUMBBELL-κB decoy was confirmed in the same way as described in our previous report^10^, i.e. by significant (ca. 50%) decrease of luminescence in cells transfected with the unplatinated DUMBBELL-κB, whereas the transfection with scrambled DUMBBELL lacking the кB site had no significant effect on luciferase activity in cells. When the cells were transfected with the DUMBBELL-κB globally modified by BBR3464 at r_b_ = 0.021, the decoy activity was inhibited by 73% (Fig. 9B). These data suggest that BBR3464 strongly inhibited cellular NF-кB binding to the decoy oligonucleotide. As indicated in Fig. 9B, BBR3464 was found to be the more effective in inhibiting decoy activity when compared with cisplatin, whereas modification of decoy oligonucleotide by transplatin had no significant effect on the decoy activity^10^.

## Discussion

In the present investigation, electrophoretic mobility shift assay and SPR spectroscopy were used to examine the binding affinity of NF-κB proteins to DNA duplexes containing κB site which was damaged by DNA adducts of antitumor trinuclear platinum(II) complex BBR3464. The effects of these DNA adducts were compared with those of less cytotoxic cisplatin and ineffective transplatin. We observed a strong inhibitory effect of DNA adducts of BBR3464 on the binding affinity of native NF-κB proteins or NF-κB proteins reconstituted from purified p50 and p65 proteins. Inhibition of the formation of the complex between NF-κB proteins and DNA duplexes containing κB site by DNA adducts of cisplatin or antitumor-inactive transplatin was significantly less, being least effective for transplatin. Examination of the association and dissociation phases of SPR sensorgrams yielded the kinetic parameters responsible for the decrease of affinity of NF-κB proteins to platinated κB sites in DNA. These parameters indicated that this decrease was related mainly to variation of the association step. Importantly, the effect of modification of the кB site by BBR3464, cisplatin or transplatin on the DNA binding activity of NF-кB proteins in cells was also studied employing the decoy strategy. The results of these *in cellulo* experiments were in full agreement with the conclusions drawn on the basis of the experiments performed in cell-free media with recombinant p50 or p65 proteins or with whole cell extracts prepared from the stimulated cells in which NF-κB signal transduction pathway was activated.

The κB sites in DNA contain several sites at which adducts of platinum complexes tested in the present work are formed. In the κB sites, the platinum complexes form adducts, which disturb DNA secondary structure. We suggest that the result of these perturbances is that the tuned steric fit required for the formation and stability of the complex of NF-κB protein with the κB site cannot be attained so that NF-κB protein binds to its platinated consensus sequence in DNA (κB site) with a reduced affinity. Transplatin, cisplatin and BBR3464 form discrete adducts in DNA which disturb DNA secondary structure to a different extent^21^,^30^. For instance, ineffective transplatin forms adducts in DNA which induce relatively subtle structural perturbations in DNA^31^, which apparently have no substantial effect on the formation of the complex of NF-κB protein with the κB site. On the other hand, major adducts of cisplatin disturb DNA conformation more^3^ and consequently, the inhibition of binding of NF-κB proteins to the κB sites in DNA was markedly more efficient for adducts of cisplatin than for those of transplatin. DNA adducts of trinuclear BBR3464 are mainly long-range delocalized CLs between guanine residues so that conformational distortions induced by these long range CLs extend over a considerably larger part of the κB sites than those induced by major CLs of mononuclear cisplatin^21^. In addition, the central tetraamine linker of the long-range interstrand CLs of BBR3464 (the role of which predominates in the antitumor effects of BBR3464^23^) is situated in or very close to the minor groove of DNA^32^,^33^. This location of the linker could also sterically block the binding of the NF-κB protein to DNA since it could restrict narrowing of the minor groove required for NF-κB protein binding^14^. Thus, a plausible explanation of the higher efficiency of DNA adducts of BBR3464 to inhibit binding of NF-κB protein to its κB site in comparison with the adducts of cisplatin may lie in the enhanced extent of conformational perturbations induced in DNA by the trinuclear platinum complex.

These *in vitro* and *in cellulo* studies thus demonstrate that affinity of NF-κB proteins to DNA modified by the bifunctional Pt^II^ complexes tested decreases with their growing cytotoxic effectiveness. Our findings suggest that mechanisms of toxicity of antitumor platinum complexes in tumor cells may involve interference with NF-κB signaling pathways leading to its antiapoptotic effects. This interference might be connected with efficiency of platinum drugs to form on DNA consensus sequences of these transcription factors the adducts which effectively distort conformation DNA. The results of this work are consistent with the hypothesis that a control of protein recognition and pathways leading to cell death by properly designed DNA adducts may be an interesting approach to predict more efficient anticancer metallodrugs. The confirmation of this hypothesis and its full generalization will require to evaluate a larger series of platinum(II) complexes.

It can be expected that in a near future new transition metal-based compounds will be designed, synthesized and tested for their biological effects, which will be able to form on DNA the adducts distorting conformation of this nucleic acid still more than DNA adducts of conventional antitumor platinum compounds. It is also reasonable to predict that DNA adducts of these new complexes will inhibit binding of NF-κB protein to its κB site more in comparison with the adducts of platinum compounds so far tested and concomitantly antitumor effects of these new complexes will be potentiated.

## Methods

### Chemicals

**Figure 1:**
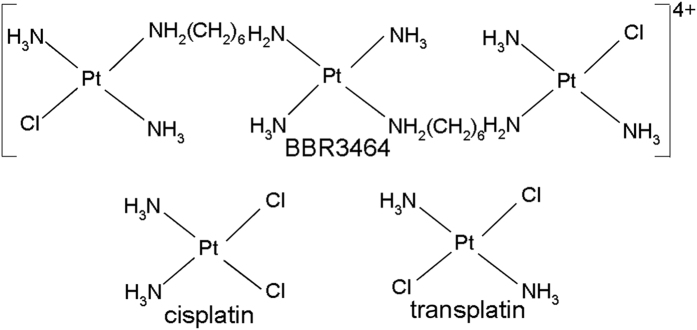
Structures of platinum compounds.

**Figure 2:**
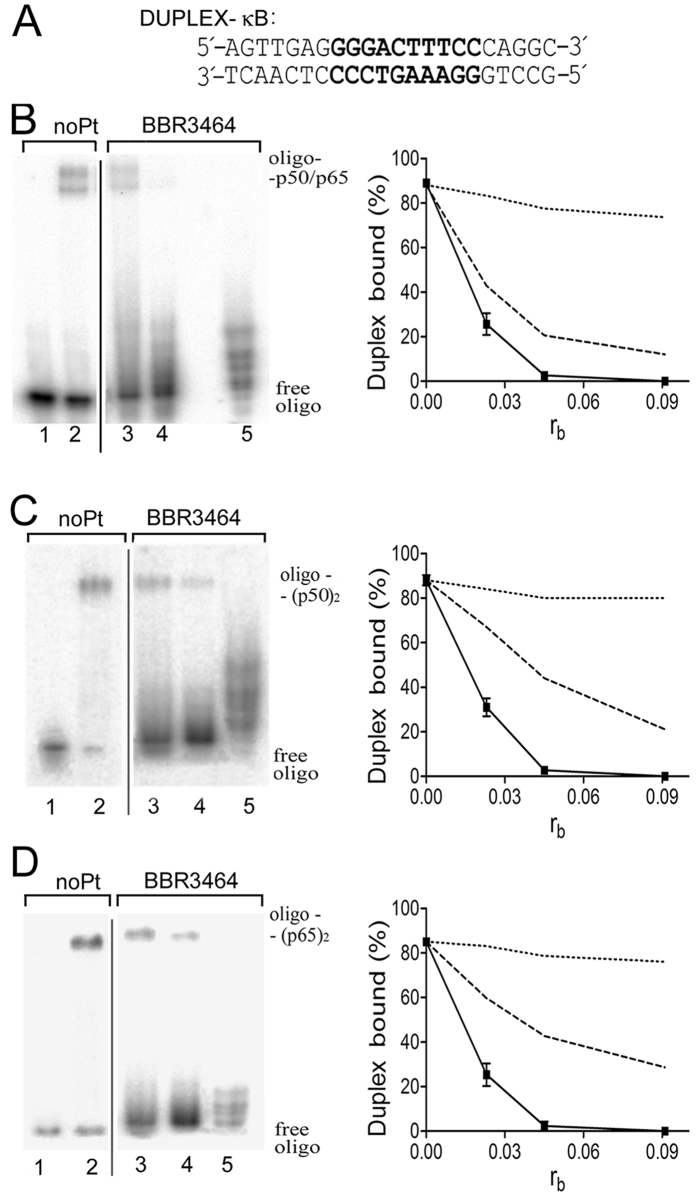
Binding of NF-кB proteins to the DNA duplex containing the кB site. (**A**) The nucleotide sequence of the 22-bp oligodeoxyribonucleotide duplex containing κB site (DUPLEX-κB). The bold letters in the sequence indicate the κB recognition sequence. Left panels in Fig. 2B–D. Binding of p50/p65 heterodimer and p50/p50 and p65/p65 homodimers to the DUPLEX-κB containing the кB site ((**B–D**), respectively). The panels show autoradiograms of the EMSA experiments showing the binding of p50/p65 heterodimer (Fig. 2B), p50/p50 (Fig. 2C) and p65/p65 (Fig. 2D) homodimers to the DUPLEX-κB. Lanes 1 and 2, non-modified duplex; lanes 3–5, duplex globally modified by BBR3464 at r_b_ = 0.023, 0.045, or 0.091, respectively. The gel mobility shift assay was performed as described in the section Materials and Methods; concentration of the oligonucleotide duplex was 1 nM and the concentrations of p50/p50, p65/p65 and p50/p65 were 10, 15 and 15 nM, respectively. Right panels in Fig. 2B–D. Plots of the amount of the DUPLEX-κB modified by BBR3464 (full line), cisplatin (dashed line) or transplatin (dotted line) in complex with p50/p65 heterodimer (Fig. 2B), p50/p50 (Fig. 2C) and p65/p65 homodimers (Fig. 2D) on r_b_; the data for cisplatin and transplatin were taken from ref. 10. Data are the mean ± SD obtained from three different experiments.

**Figure 3:**
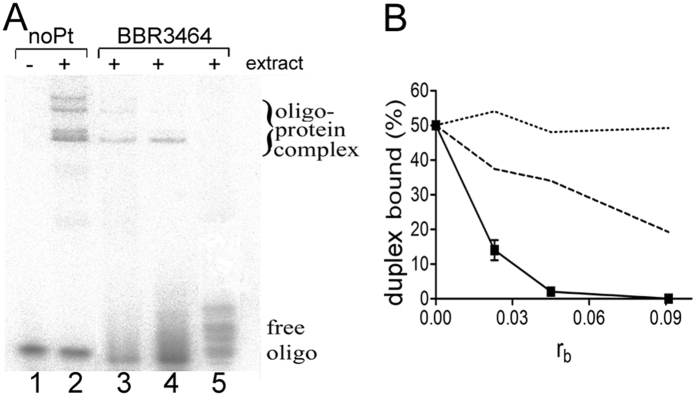
Binding of native NF-кB to the 22-bp oligonucleotide duplex containing кB recognition site (DUPLEX-κB). (**A**) Autoradiogram of EMSA experiment showing binding of native complex of NF-кB from whole cell extract stimulated by Il-1α to the duplex containing кB site. Lanes: 1 and 2, unmodified duplex; 3, 4, and 5, duplex globally modified by BBR3464 at r_b_ = 0.023, 0.045, or 0.091, respectively. (**B**) Quantitative evaluation of the amount of native NF-кB in complex with the oligonucleotide duplex modified by BBR3464 (full line), cisplatin (dashed line) or transplatin (dotted line) at various r_b_; the data for cisplatin and transplatin were taken from ref. 10. Data are means ± SD from three different independent experiments.

**Figure 4:**
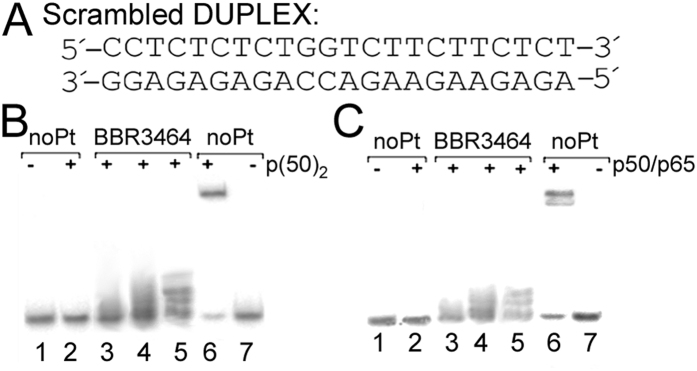
Binding of NF-кB proteins to the Scrambled DUPLEX. (**A**) Nucleotide sequences of the 22-bp oligodeoxyribonucleotide duplex lacking κB site (Scrambled DUPLEX). (**B,C**) Autoradiograms of the EMSA experiments showing the binding of NF-кB p50/p50 homodimer (panel B) or p50/p65 heterodimer (panel C) to the Scrambled DUPLEX. Lanes 1 and 2, non-modified duplex; lanes 3–5 duplex modified by BBR3464 at r_b_ = 0.023, 0.045, or 0.091, respectively. A gel mobility shift assay was performed as described in Materials and Methods; concentrations of the Scrambled DUPLEX was 1 nM and the concentrations of p50/p50 and p50/p65 were 10 and 15 nM, respectively. As a control, binding of p50/p50 and p50/p65 to the nonplatinated duplex containing consensus кB site was analyzed in parallel (lanes 6 and 7).

BBR3464 (nitrate salt) (Fig. 1) was prepared as described previously^33^,^34^. Cisplatin, transplatin, Sephadex G-50 and NP-40 were purchased from Sigma (Prague, Czech Republic). Recombinant p50 protein was kindly provided by Prof M. Vasak, University of Zurich. Recombinant p65 protein, HeLa whole-cell extract (IL-1α stimulated) and antibodies against the p50 and p65 subunits of NF-κB were from Active Motif (Rixensart, Belgium). Poly(dI-dC).poly(dI-dC) was purchased from Biochemicals, Inc. (Milwaukee, USA). The synthetic oligodeoxyribonucleotides and biotinylated oligodeoxyribonucleotides purchased from VBC Biotech (Vienna, Austria) were purified by HPLC as described previously^35^. Acrylamide, bis(acrylamide) and dithiothreitol (DTT) were from Merck KgaA (Darmstadt, Germany). Sodium dodecyl sulfate (SDS), penicilin, hygromycin B, and streptomycin were from Serva (Heidelberg, Germany). [γ-^32^P]ATP was from Amersham (Arlington Heights, IL). Streptavidin coated SA sensor chips were from BIAcore AB (Uppsala, Sweden). T4 DNA ligase and Exonuclease III were from New England Biolabs (Beverly, MA, USA).

### Cell line

Stable human NF-κB-RE-*luc2*P HEK293 cell line was obtained from Promega (Madison, WI, USA). Cells were grown at 37 °C with 5% CO_2_ in DMEM supplemented with fetal bovine serum (10%), hygromycin B (50 μg mL^−1^), penicillin (100 U mL^−1^) and streptomycin (100 μg mL^−1^).

### Oligonucleotide probes

If not stated otherwise, the top strands of the duplexes used in the electrophoretic mobility shift assay (EMSA) experiments were radioactively labeled at their 5′-end (γ-^32^P ATP; T4-polynucleotide kinase). The unincorporated γ-^32^P ATP was disposed using Sephadex G-50 fine columns. To anneal the radiolabeled top strands with equimolar amounts of corresponding bottom strands the samples were heated to 65 °C for 10 min in a solution containing Tris.HCl (50 mM, pH 7.0), MgCl_2_ (10 mM), and DTT (1 mM), and then allowed to cool down to 4 °C for 4 h. It was verified by loading the samples onto 15% native PAA gels that they contained no non-annealed single-stranded oligonucleotide. It is to be understood that in the following text, the molar concentrations of the single-stranded oligonucleotide or oligonucleotide duplexes are related to the oligomers or double-stranded molecules, respectively (not to the monomer content).

### Platination reactions

Oligonucleotide duplexes were platinated in NaClO_4_ (10 mM) at 37 °C for 24 h in the dark, followed by precipitation by ethanol to remove free, unbound platinum. The levels of modification (average number of platinum molecules coordinated per nucleotide residue) were determined using FAAS and UV spectrophotometry.

### Gel mobility shift assay

EMSAs were performed as described in several previously published papers^10^,^36^,^37^. Briefly, the total volume of 20 μL contained Tris.HCl (10 mM, pH 7.5), NaCl (50 mM), DTT (1 mM), EDTA (1 mM), bovine serum albumin (BSA) (10 mgmL^−1^), NP-40 (1%), glycerol (5%), poly(dI-dC).poly(dI-dC) (4.1 × 10^−5^ M related to the phosphorus content), radiolabeled oligonucleotide duplex (non-modified or platinated with BBR3464, 0.02 pmol), purified proteins p50/p50 (0.2 pmol), p65/p65 (0.3 pmol), heterodimer p50/p65 (0.3 pmol) or IL-1α stimulated HeLa whole-cell extract (1 μL, ∼2 μg of proteins). To form the p50/p65 heterodimer equal amounts of p50 and p65 were incubated at 37 °C for 60 min (final protein concentration of 0.2 pmolμL^−1^). Following the incubation at 20 °C for 45 min, the samples were loaded onto 6% PAA gels in 0.5xTris-borate-EDTA (prerun for 1 h at 300 V; 4 °C). The electrophoresis was run under the same conditions for 1.5 h and the gels were dried and exposed to a molecular imaging plate. The autoradiograms were recorded using a FUJIFILM bioimaging analyzer and processed with AIDA image analyzer software.

The following equation was employed to calculate the percentage of DNA bound to protein^10^:

Oligonucleotide duplex bound (%) = [protein-DNA complex/total DNA] × 100% = [protein-DNA complex/(free DNA + protein-DNA complex)] × 100%

### Preparation of biotinylated constructions for Surface Plasmon Resonance (SPR) measurements

**Figure 5:**
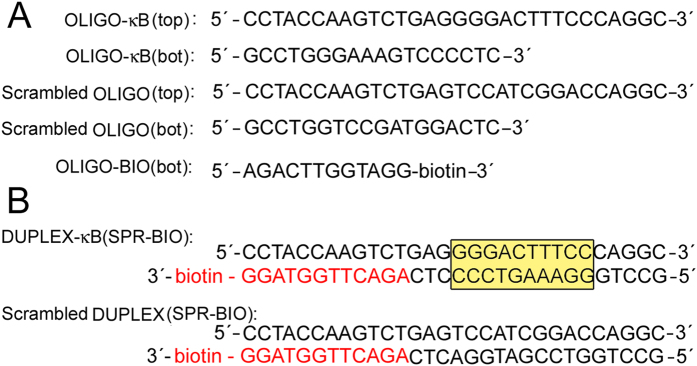
(**A**) Sequences and designation of oligodeoxyribonucleotides and DNA duplexes used in SPR experiments. OLIGO-κB(top), 30-nt oligonucleotide containing κB-site. OLIGO-κB(bot), 18-nt oligonucleotide containing κB-site complementary to OLIGO-κB(top). Scrambled OLIGO(top), 30-nt oligonucleotide containing no κB-site. Scrambled OLIGO(bot), 18-nt oligonucleotide containing no κB-site complementary to scrambled OLIGO(top). OLIGO-BIO(bot), 3′-biotinylated 12-nt oligonucleotide complementary to both OLIGO-κB(top) and scrambled OLIGO(bot). (**B**) Constructs of duplexes for SPR experiments. DUPLEX-κB(SPR-BIO), biotinylated 30-bp duplex containing κB-site (yellow). Scrambled DUPLEX(SPR-BIO), biotinylated 30-bp duplex containing no κB-site.

**Figure 6:**
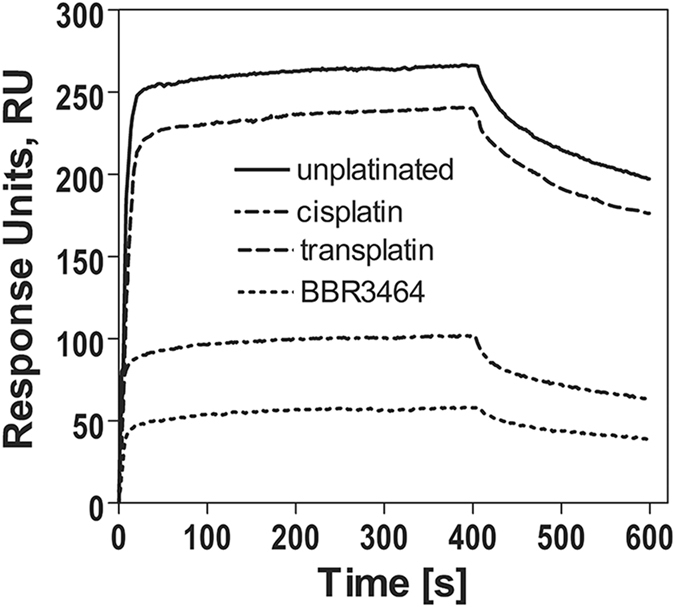
Typical SPR sensorgrams showing the interactions between p50/p50 homodimer and immobilized DUPLEX-κB(SPR-BIO) unplatinated or modified by BBR3464, cisplatin or transplatin. The concentration of p50/p50 homodimer was 100 nM.

**Figure 7:**
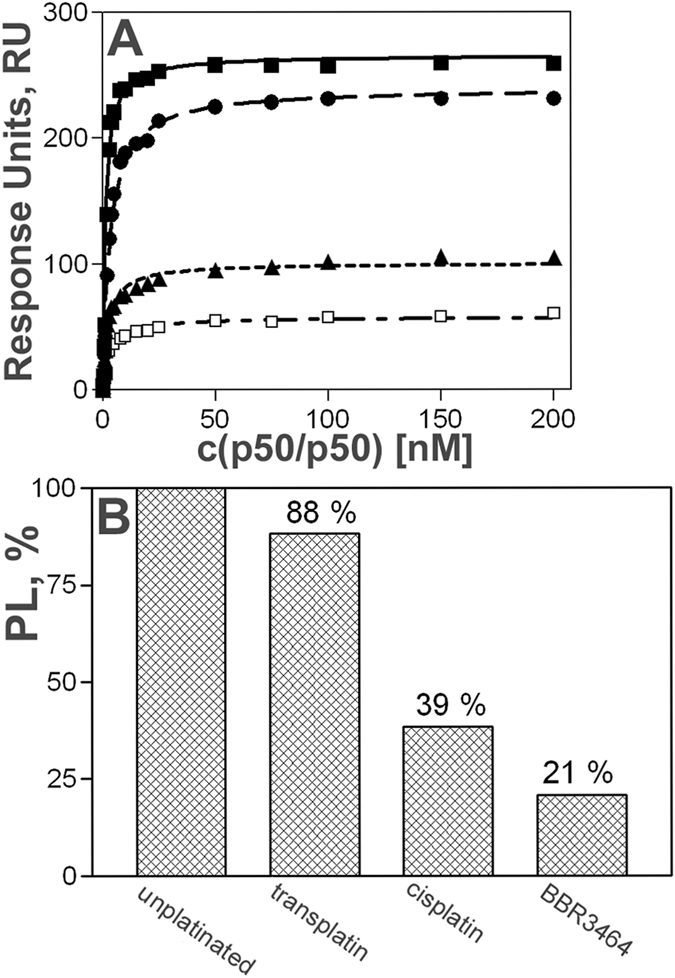
Steady-state analysis of the binding of p50/p50 homodimer to immobilized DUPLEX-κB(SPR-BIO) unplatinated or modified by BBR3464, cisplatin or transplatin from SPR experiments. (**A**) After blank subtraction, RU values at 400 s were read and plotted against protein concentration. Close squares – unplatinated DNA; close circles – transplatin modified DNA; close triangles – cisplatin modified DNA; open squares – BBR3464 modified DNA. (**B**) Comparison of plateau level values (PL) for DUPLEX-κB(SPR-BIO) unplatinated or modified by transplatin, cisplatin and BBR3464. The value of PL obtained for unplatinated duplex was taken as 100%.

**Figure 8:**
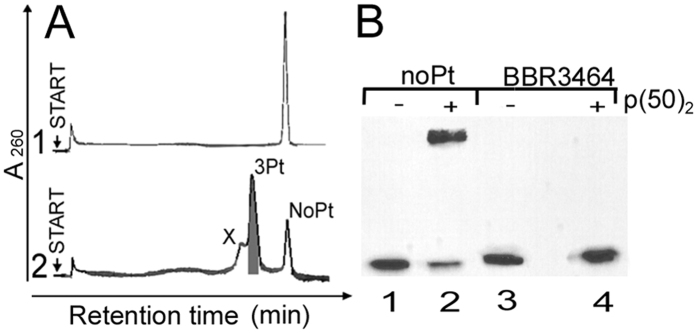
(**A**) The HPLC profiles of nonplatinated (curve 1) or BBR3464-modified (curve 2) bottom strands of the oligonucleotide containing κB site (DUPLEX-κB, for its nucleotide sequence, see Fig. 2A). (**B**) Autoradiogram of the EMSA gel showing a binding of NF-кB p50/p50 homodimer to the кB site containing oligonucleotide duplex carrying single adduct of the BBR3464. Lanes 1 and 2, unplatinated duplex; lanes 3 and 4, duplex modified by only one adduct of BBR3464 in the absence of unplatined duplexes.

**Figure 9:**
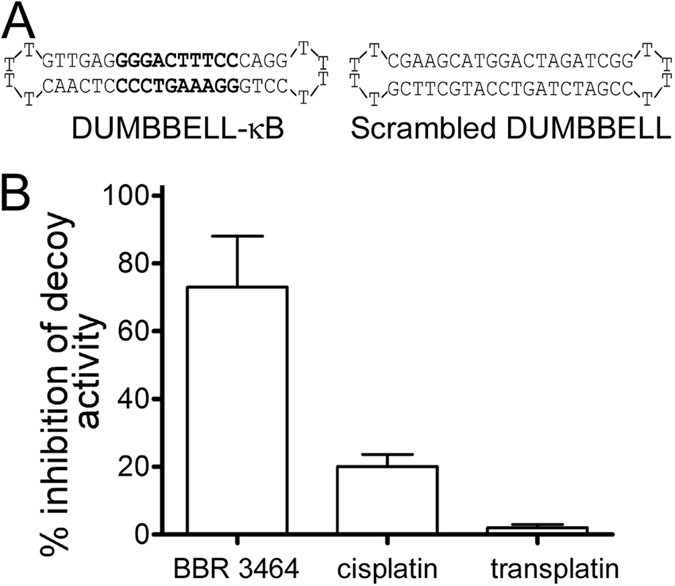
Differential inhibition of dumbbell decoy activity by DNA adducts of BBR 3464 as compared with cisplatin and transplatin in HEK293-NF-кB-luciferase reporter cell line. The percentage of inhibition of the specific decoy activity was calculated by measuring, for each experiment, the difference between luciferase activities obtained with cells transfected with platinated DUMBBELL-кB and nonplatinated DUMBBELL-kB divided by the difference between luciferase activities obtained with cells transfected with nonplatinated scrambled and specific (κB-site containing) DUMBBELL decoy oligonucleotides. Data represent the mean ± SD obtained from triplicate wells and are representative of at least three independent experiments. Data for ciplatin and transplatin were taken from ref. 10.

The oligonucleotide duplexes were prepared as schematically shown in Fig. 5. Equimolar amounts of top strand (30-mer) and bottom strand (18-mer) were annealed in NaClO_4_ (0.02 M) at 65 °C for 10 min and then allowed to cool down to 4 °C for 4 h. These duplexes, which contained no biotinylated part of the bottom strand, were then incubated with BBR3464, cisplatin or transplatin in the ratio of one molecule of the platinum complex per one molecule of the duplex for 24 h. The platinated duplexes were subsequently exhaustively dialyzed to remove any residual platinum complexes which were not coordinatively bound to the duplex. The platinated duplexes were annealed with 3′-end biotinylated bottom strand (12-mer) in Tris.HCl (10 mM) and NaCl (100 mM) for 4 h at 4 °C. An excess of duplex was used at this step so that no biotinylated single-stranded oligonucleotides remained in the mixture. This precaution was used to prevent streptavidin coated chips to bind to single-stranded biotinylated oligonucleotides.

### SPR spectroscopy

SPR measurements were performed on a BIAcore 3000 at 20 °C using a streptavidin coated chips and oligonucleotide DNA duplexes containing a 3′-biotin on the bottom strand. Biotinylated oligonucleotide constructions were bound to the streptavidin-coated SA sensors by injecting Tris.HCl (10 mM, pH 7.5), EDTA (3 mM), NaCl (0.63 M), glycerol (2%), surfactant P-20 (0.005%) and 1 nM biotinylated duplexes at a flow rate of 10 μLmin^−1^. The injection was stopped when the amount of bound DNA corresponded to the signal increase of 50 RU. The overall increase in signal for all samples ranged from 50 to 52 RU. The protein p50 was injected at a flow rate of 20 μLmin^−1^ in running buffer [Tris.HCl (10 mM, pH 7.4), NaCl (150 mM), glycerol (10%), DTT (3 mM), EDTA (0.2 mM) and P-20 (0.005%)]. Two washes of 10 μL of running buffer containing SDS (0.04%) were used to regenerate the surface after each injection of p50. Binding of each duplex was assayed at concentrations of p50 ranging from 0 to 200 nM. To obtain kinetic parameters, late association phases and early dissociation phases of the resulting sensorgrams were fit to the Langmuir binding model using BIAevaluation software (version 3.1). After blank subtraction RU values at 400 s (where the response was maximal obtained at the plateau level) were read and plotted against protein concentration.

## Additional Information

**How to cite this article**: Brabec, V. *et al*. Inhibition of nuclear factor kappaB proteins-platinated DNA interactions correlates with cytotoxic effectiveness of the platinum complexes. *Sci. Rep.* **6**, 28474; doi: 10.1038/srep28474 (2016).

## Figures and Tables

**Table 1:** Kinetic and thermodynamic parameters for the complexes formed between DUPLEX-κB(SPR-BIO) unplatinated or modified by transplatin, cisplatin or BBR3464 and p50/p50 homodimer obtained from SPR experiments^a^.

	*k*_on_ (M^−1^s^−1^)^b^	*k*_off_ (s^−1^)^c^	RU_MAX_	*K*_D_ (nM)^d^	Δ*G*^0^_310_(kJmol^−1^)^e^
unplatinated	6.26 × 10^5^	1.23 × 10^−3^	266	1.96	−51.7
transplatin	4.94 × 10^5^	0.98 × 10^−3^	240	1.99	−51.6 (0.1)
cisplatin	4.20 × 10^5^	1.22 × 10^−3^	101	2.38	−51.1 (0.6)
BBR3464	3.04 × 10^5^	1.21 × 10^−3^	58	3.99	−49.8 (1.9)

^a^Five different concentrations (10–200 nM) of p50/p50 homodimer were analyzed.

^b^*k*_on_ denotes the association rate constant.

^c^*k*_off_ denotes the dissociation rate constant.

^d^*K*_D_ denotes equilibrium dissociation constant.

^e^Δ*G*^0^_310_ denotes the Gibbs free energy change for complex formation at 310 K [Δ*G*^0^_310_ = -RT ln *K*_D_, where T is the temperature in Kelvin and R is the universal gas constant (8.314472 J K^−1^ mol^−1^)].
